# Public awareness and perception of Ghana’s restrictive policy on fatty meat, as well as preference and consumption of meat products among Ghanaian adults living in the Kumasi Metropolis

**DOI:** 10.1186/s40795-018-0209-z

**Published:** 2018-02-05

**Authors:** Reginald A. Annan, Charles Apprey, Nana Kwasi Oppong, Vanessa Petty-Agamatey, Laudina Mensah, Anne Marie Thow

**Affiliations:** 1 Department of Biochemistry and Biotechnology, College of Science, Kwame Nkrumah University of Science and Technology, Private Mail Bag, University Post Office, Kumasi, Ghana; 2 Menzies Centre for Health Policy, School of Public Health, University of Sydney, Camperdown, Australia

**Keywords:** Fatty meat, Policy, Awareness, Consumption

## Abstract

**Background:**

The nutrition transition, currently observed across the world is driven by trade liberalization, urbanization and more sedentary lifestyles. Ghana implemented a restrictive policy to limit the availability and access to fatty meat in the 1990s. This paper sought to determine public awareness and perception of the policy’s enforcement and impact, as well as the general attitude towards fatty meat, preference and consumption of meat types.

**Method:**

A cross-sectional qualitative study was carried out among 377 adults, aged ≥18 years, living in Kumasi Metropolis in the Ashanti Region, the second largest city in Ghana. An interviewer administered structured questionnaire and a food frequency questionnaire were used for data collection. Body composition parameters were determined with OMRON body composition analyser and blood pressure was measured with a digital sphygmomanometer.

**Results:**

Majority of respondents were females (62.9%), aged 18–35 years old (72.1%) and were labourers, traders or teachers (52.5%). Mean Body Mass Index of participants was 25.4 kg/m^2^ and percent body fat was 30.4%. Over half (58.9%) of the participants were aware of, and most (81.2%) supported the restrictions, although majority (57%) thought there was low public awareness and less than 15% felt the restrictions were well enforced. About 59.4% believed the restriction could improve health, prevent disease and reduce deaths in the long-term. Two thirds (67.1%) of the participants considered the fat content of the meat they bought and related fat to health problems (38.5%) and obesity/stroke (7.4%). Local meat products (meat produced in Ghana) were more preferred due to taste, freshness and healthiness than imported meat, but imported meat types (meat imported from other countries) were more available (56.5%), cheaper (69.5%) and regularly consumed than the local types.

**Conclusion:**

There was a good level of public awareness, strong support and positive attitude towards Ghana’s restrictive food standards on meat. Although preference for local meat type was greater, imported meat was more consumed due to cost and availability. Policies which limit access to, and availability of ‘unhealthy’ food should be implemented and enforced to improve the food environment in order to help address the growing obesity and non communicable disease trend.

## Background

Many countries in sub-Saharan Africa including Ghana are currently faced with a double burden of malnutrition. On one end of the spectrum is undernutrition and related communicable diseases. The other end of the spectrum shows increasing prevalence of over nutrition and diet-related chronic diseases, and their associated deaths. Overweight and obesity are also common and it is possible to find overweight/obese adults with micronutrient deficiencies [1]. Cardiovascular diseases (CVD), including coronary heart disease (CHD), stroke and heart failure, are associated with 16.7 million deaths in the world every year [2], with 80% of all CVD deaths occurring in developing countries [3]. In Ghana, the national prevalence of hypertension was estimated at 28.7% [4]. Heart diseases and diabetes are on the rise in the region and figures for hypertension show levels ranging from 3% in rural areas to more than 30% among urban dwellers [4]. Over 80% of the diabetics are of type 2, the form associated with obesity and physical inactivity. The WHO predicts increases in deaths from chronic diseases in low- and middle-income countries such as Ghana by 2030 [3].

Generally, dietary patterns characterized by high intake of fatty foods and refined carbohydrates, and a low consumption of fruits and vegetables are commonly reported to be strongly associated with metabolic risk factors for Cardiovascular Diseases (CVDs) and other chronic diseases [1, 5]. Referred to as the nutrition transition, increased sedentariness and a shift in patterns of dietary intake towards more westernised foods [6], characterised by increased intake of saturated fat, salt and refined foods and decreased fruits, vegetables and fibre intake have been reported in many developing countries [7, 8]. The nutrition transition has also contributed to an epidemiological transition characterised by increasing incidence of diet-related chronic diseases [9]. In Ghana, where the percentage of adults who consume sufficient amounts of fruits and vegetables is less than 5%, increased consumption of foods high in fats, sugars and salt is a major contributor to the increasing prevalence of CVD currently observed [10]. Thus, although the threat of undernutrition remains, the observed trend shows that diet-related chronic disease incidence and its consequences require national interventions.

The changing pattern of dietary intake and lifestyle is spurred by urbanization, liberalisation of market and trade (especially in foods), physical inactivity and demographic shifts [11–13]. Consequently, international institutions, non-government agencies and academics have recommended the use of trade policy tools in food policies designed to improve the healthfulness of the food supply [14, 15]. Recommendations include the use of food composition standards, reducing import tariffs on fruits and vegetables, maintaining high tariffs on unhealthy food imports and reducing imports of unhealthy foods.

The importation of cheap fatty cuts of meat has been identified in Africa and the Pacific as a contributor to both poor health (due to high fat intakes) and agricultural under-development [16, 17]. Research in the Pacific has documented policies banning imported fatty meat products [18]. However, an ongoing challenge has been the development of non-discriminatory food policy tools that are consistent with international trade commitments. In the early 1990s, the Government of Ghana implemented an innovative policy to limit the amount of fat in meat products (both imported and domestically produced) in order to protect human health [19]. The policy sets the standards for fat content in meat at 25% or less for pork, mutton and beef, and 15% or less for poultry. To date, however, the only products banned under the standard have been imported turkey tails and chicken feet. In August 2013, a study to evaluate the process, outcomes and lessons learned from this innovative policy was carried out. Case study research and policy analysis methodologies were used to evaluate the policy, which enabled in-depth assessment of the policy process and outcomes in Ghana, while enabling maximization of the usefulness of the case study for policy learning in other contexts. Based on the ‘health policy analysis triangle’, data on the process (agenda-setting, policy development, implementation), context, content and actors (local and international) for the policy as well as the policy outcomes were collected [20].

Findings of this evaluation showed that the policy had led to reduced availability of ‘low quality’ meats, especially turkey tails and chicken feet, which were mostly imported in the Ghanaian food supply [20]. It was expected that the reduction in ‘low quality’ meat availability would lead to consumption of ‘healthier’ meat types, especially the locally-produced ones, since these were deemed to have lower fat content and formed part of the context for the implementation of the policy. However, questions regarding public awareness and perception of this policy, in terms of its implementation, enforcement and impact, as well as general attitude towards fatty meat, preference for, and consumption of different meat types remained unanswered. Little is known about the contribution of fatty meat to the Ghanaian diet and the landscape, including the extent to which fatty meat is still a problem, 20 years after the implementation of the standards on fatty meats. The aim of this paper is to determine public awareness, perceptions and attitudes towards the policy’s implementation, enforcement and impact. It also sought to assess the general attitude, preference and consumption of meat types by Ghanaian adults.

## Methods

The policy evaluation study was carried out in August 2013, using in-depth assessment of the policy process and outcomes in Ghana, including agenda-setting, policy development, implementation, context, content and actors (local and international) for the policy as well as the policy outcomes [20]. Twenty-seven (27) semi-structured interviews were conducted with policy makers in Trade, Health and Agriculture; Implementers in Ghana Standards Authority, Ghana Food and Drugs Authority, Animal Extension and Customs Excise and Preventive Service (CEPS); Traders and importers; and Agricultural producers. Also, broad contextual questions regarding the historical trade policy context, changes in food prices and agricultural production, and the perceived effect of food imports on agricultural production were delved into [20].

### Study design and population

Data for this study were collected between February to April 2015 using a cross sectional qualitative design among adults, aged 18 years and above living in suburbs of Kumasi Metropolis, the second largest city of Ghana. This study sought to assess participants’ awareness of the policy, what they thought the general awareness of the Ghanaian public was with regards to the policy, whether they supported the policy, and their thoughts about how well the policy has been enforced and its long-term impact. Participants’ attitudes towards fatty meat in general, as well as preference for and consumption of different meat types were also assessed.

### Sample size

The sample size equation [Sample size = Z_1-**α/**2_^2^p(1-p)/d^2^] [21] for cross sectional study (qualitative variable) was used to determine the sample size. Where, Z_1-**α/**2_ is standard normal variable at 5% type 1 error (*p* < 0.05), which is 1.96, p is the expected proportion of participants aware of the restriction set at half of the population, 50% or 0.5 (as this was unknown) and d is the precision/absolute error of 5% and at type 1 error of 5%. Using the above formula, a sample size of 384 was calculated for the study, although data was eventually collected from 377 participants.

### Participants recruitment

Three hundred and seventy-seven (377) participants, aged 18 years and above living in 9 suburbs (Atonsu Agogo, Anloga, Atonsu, Ayigya, Ayeduase, Ayeduase Newsite, Kotei, Kwadaso, and Oforikrom) of Kumasi were recruited by final year undergraduate students from the Kwame Nkrumah University of Science and Technology (KNUST) trained for this study. The suburbs, although purposively selected, were all big towns in Kumasi, and were chosen to give a good spread of participants from the Metropolis. In each suburb, study enumerators went from house-to-house to recruit participants. Participants were recruited on first-come-first-service basis from house-to-house visits until at least 30 participants were recruited. The selection of houses was not systematic nor random since enumerators could enter any house upon entering the suburb and move to the next house afterwards. If a house had no adult available at the time of visit, the next house was entered. The direction of the next house from the preceding one was completely dependent on the enumerators. For each house entered, adults living in the house got the aims, objectives and other information about the survey explained to them, after which they were asked to volunteer. Only one adult was chosen from each house and it was usually the first person who volunteered to take part. Apart from the 30 participants selected from each suburb of the 9 suburbs by house-to-house visits, some participants were met on the road side in the suburbs and asked to participate. Some churches and workplaces were visited and people asked to volunteer until the required sample size was attained.

### Data collection

A validated structured questionnaire was adapted for this study, which had been developed and used for assessing consumer responses to the turkey tail ban in Samoa [18]. The questionnaire was interviewer administered and apart from personal information, had close-ended questions regarding awareness of the policy, support for the policy, attitude and preference for meat types, comparing imported versus local meat types. Open-ended questions were used to assess participants’ perception of the outcome and impact of the restriction. The questionnaire also included a Food Frequency Questionnaire (FFQ). The FFQ used was based on a standard layout for FFQs but was adapted to suit the local food (meat and meat products) environment of Kumasi. The FFQ used had 7 meat types (local versus imported beef, chicken, turkey tail, pork, mutton, goat meat and processed meat) and 5 options for frequency of consumption: Weekly, Fortnightly, Monthly, Occasionally and Never. The entire questionnaire and study instruments were pretested by interviewers among adults living near the University campus as part of training for data collection. The study instruments were amended following the pretest.

Assessment of body composition and blood pressure were done using the OMRON body composition analyser and a digital sphygmomanometer respectively. For the body composition assessment, participants’ height in centimeters were first measured using a SECA stadiometer and then the heights, age and gender were entered into the analyser for the assessment of body composition. The purpose of this was to know the status of these parameters among participants.

### Data analysis

The data were analysed using SPSS software, version 17.0.1 (SPSS Inc., Chicago IL, USA). Statistical significance was set at *p* < 0.05 for all analyses. Data from the food frequency questionnaire were used to describe and compare consumption pattern of imported versus local meat types and products among the participants. All other analyses were done using simple frequencies and percentages and presented as tables.

## Results

### Description of respondents

Of the 377 study participants, 140 (37%) were males and 237 (63%) were females (Table 1). A larger proportion of the respondents (72%) were within 18–35 years age range, followed by 36–50 years (23%) and above 50 years old (5%). There were varied occupational activities among the respondents but just over a third were labourers, 18.3% were traders and 10.9% were teachers. Mean BMI of the respondents was 25.4 kg/m^2^, and mean body fat was 30.4%, while mean systolic and diastolic blood pressure of participants were 123.5 and 77.25 mmHg respectively**.**

**Table 1:** Personal information of respondents

Variable	Frequency	Percentage
Age (years)		
18–35	272	72.1
36–50	86	22.8
> 50	19	5
Gender		
Male	140	37.1
Female	237	62.9
Occupation		
Trader	69	18.3
Farmer	8	2.1
Teacher/lecturer	41	10.9
Health worker	19	5
Labourer	129	34.2
Office worker	28	7.4
Student	21	5.6
Unemployed	32	8.5
Engineer	6	1.6
Sales girl/boy	14	3.7
Security personnel	1	0.3
Drivers	6	1.6
Body Composition		
BMI	25.4	0.4
Percentage Body Fat	30.43	0.95
Visceral Fat	6.25	0.45
Percentage Muscle mass	31.35	0.5

### Public awareness and perception about restriction

Two hundred and twenty-two (59%) respondents were aware of the restrictions on fatty meat importation (Table 2). Of those aware of the restriction, majority heard it from radio broadcast 101 (46%), Television (20%) and word of mouth (10%). Close to 13% heard from two of the named avenues and about 3% from all three avenues. Over half of the respondents (57%) thought the public were not aware of the restrictions. Most of the respondents (whether aware or not aware) (306, 81.2%) thought the restrictions are necessary and/or supported the restrictions. Of these, the top reason given was that fatty meat causes diseases and health problems (50%) and that the restriction is needed to check meat quality (22.5%). A small proportion said the restriction was good for the nation (7.2%) and is needed help to protect local industry (9.7%). Of the 38 (10.1%) respondents who thought the restrictions were not necessary, most (87.5%) said there was no information about the need for restriction, others said local meat is expensive (28.6%), while others asserted that the government should not control what people choose to eat (20%). Less than 15% felt the restrictions were being enforced.

**Table 2:** Perception, awareness and enforcement of restrictions

*N* = 377	Frequency	Percentage
Are you aware of the restriction & is the public aware?		
Yes	222, 147	58.9, 39
No	155, 215	41.1, 57
How did you hear about the restriction?		
TV	45	20.1
Radio	101	46.1
Newspaper	7	3.1
Word of mouth	23	10.3
Other means	5	2.2
More than one channel	38	17.1
Do you think the restriction is being enforced or necessary?		
Yes	55	14.9
No	308	81.7
Don’t know	14	3.7
Do you think the restriction is necessary?		
Yes	306	81.2
No	38	10.2
Don’t know	32	8.5
Why is the restriction necessary? (*N* = 236)		
Fatty meat causes diseases and health problems	118	50
Restriction will help reduce deaths	14	5.9
Restriction good for the nation	17	7.2
Needed to promote local industry	23	9.7
Imported meat unwholesome due to prolong storage	11	4.7
To check the quality of meat	53	22.5

### Respondents’ attitude towards fatty meat

From Table 3, over two-thirds of respondents (253 people) said they consider the fat content of the meat they buy. Of these, the reasons given were that fat causes health problems (57.3% or 38.5% of total survey population), personal dislike (17.4%), it ensures good nutrition (11.5%), causes obesity and stroke (11.1%) and helps prevent lipid disorders (2.8%). Among those who did not consider the fat content of the meat they buy (124, 32.9%), the reasons given included ignorance (32.3%), indifference (28.2%), fat tastes good (12.9%), preference (13.7%) and affordability (7.2%).

**Table 3:** Respondents general attitude towards fatty meat

*N* = 377	Frequency	Percentage
Do you consider the fat content of the meat you buy?		
Yes	253	67.1
No	124	32.9
Why do you consider the fat content of meat you buy?		
Fat causes health problems	145	38.5
Fat leads to obesity/stroke	28	7.4
Personal dislike	44	11.7
For good nutrition	29	7.7
To prevent lipid disorders	7	1.9
Why don’t you consider the fat content of meat you buy?		
Hunger	9	2.4
Limited options	7	1.9
Indifference	35	9.3
Fat tastes good	16	4.2
Ignorance	40	10.6
Preference	17	4.5

### Access, availability and preference: Local versus imported meat and products

Comparing imported versus local meat and products, 56.5% participants said the former were more available, but 42.4% reported the opposite. However, over two-thirds of participants reported that imported meat and products were cheaper than the local ones (Table 4). For all the meat types, with the exception of pork, participants predominantly preferred the local than the imported cuts. Reasons given for preference of local meat types were better taste (41%), access/cost (20.7%), freshness (13.3%), healthiness (18.3%) and religion (13.3%).

**Table 4:** Availability, access and preference for imported versus local imported meat

Question	Percentage
Which is more available?	
Local meat and meat products	42.4
Imported meat and meat products	56.5
Missing	1.1
Which is cheaper?	
Local meat	27.9
Imported meat	69.5
Don’t know	2.7
Preference	
Beef, chicken, pork, mutton, goat	
Local	83.6, 69.5, 33.7, 58.4, 82.6
Imported	7.2, 27.3, 18.8, 2.1, 0.8
No preference	10.2, 3.2, 47.5, 39.5, 16.4

*N* = 377

### Consumption of local versus imported meat and meat products

As shown in Table 5, imported chicken was more regularly (more than once a month) consumed than local chicken (50% versus 27.5%). Imported pork and processed meat were also more regularly consumed than the local versions. The meats for which local production was more commonly consumed than the imported versions were beef (50% versus 18.6%), goat meat (41.5% versus 27.3%) and mutton (18% versus 7%). Turkey tail was hardly consumed (< 3% consumed more than once a month).

**Table 5:** Frequency of consumption of local versus imported meat and meat products

Food item	More than once a month	Once a month	Occasional & never
Imported beef	18.6	7.2	77
Local beef	50	11.9	37.7
Imported chicken	50	13.5	38.4
Local chicken	27.5	14.1	57.9
Imported turkey tail	2.4	1.3	96.4
Local turkey tail	2.2	1.3	96.6
Imported pork	16.0	5	79
Local pork	14.4	6.1	79.6
Imported mutton	7.4	4.5	88.1
Local mutton	18.3	8	73.7
Imported goat meat	10.1	7.2	83.1
Local goat meat	41.5	13.8	54.6
Imported processed meat	27.3	12.2	60.5
Local processed meat	19.9	7.4	75.9

The FFQ used had 5 categories of frequencies. In the analysis, weekly and fortnightly were combined into one category labelled more than once a month, and occasionally and never also combined into one category

### Perceptions of the effects of the fatty meat restrictions

As shown in Table 6, a third of participants were not sure of the outcome of the restriction, 23.6% thought it had had no effect whereas 19.2% thought it had reduced diseases and improved quality of life. Very few (3.7%) said the restriction had reduced importation, caused unemployment for importers (4.2%) or had led to high cost of living due to high cost of local meat (3.7%). However, when asked about the long-term effect of the restriction, close to 60% said it would improve health, prevent disease and/or reduce death. Just over 10% had no idea or thought there would be little to no effect. Whereas some participants said there would be better business and employment for local meat industries (8.3%), others said it would create unemployment for importers (5%).

**Table 6:** Participants perception of the outcome, impact and long-term effects of the restriction

Question	Frequency	Percentage
What has been the outcome of the restriction?		
No to little effect	89	23.6
Reduction on importation	14	3.7
Not sure	122	32.4
Caused unemployment for importers	16	4.2
People avoid fatty meat now	10	2.7
Local farmers are producing more	13	3.4
Reduced diseases/improved quality of life	73	19.4
Poor education about the ban	23	6.1
Unhygienic packaging of local meat	3	0.8
Expensive local meat/high cost of living	14	3.7
What do you think will be the longer-term effect of the restriction?		
It will improve health, prevent disease and/or reduce death	138	59.4
No idea/no effect	43	11.4
Importation will reduce	16	4.2
Employment for local meat sellers	13	3.4
Unemployment for importers	15	4.0
Less tax revenue for government	2	0.5
Affect Ghana’s export business	1	0.3
Local meat will be expensive	8	2.1
Policy cannot be strictly enforced	19	5.1
Higher patronage of local meat	21	5.6
Higher cost of living	10	2.7

## Discussions

Majority of the participants in this study were between 18 and 35 years old. The gender, age and occupational distribution of participants reflect the average adult Ghanaian. The average BMI puts the females as overweight and the males as normal weight. Although the females had a higher proportion of body fat, the fact that visceral fat in the males was higher reflect a higher tendency of intra-abdominal obesity in the males. Mean blood pressures were also higher in the males although both systolic and diastolic blood pressures were within the normal ranges for both gender. Combined, the study participants represented a generally healthy adult Ghanaian population.

The findings suggest that respondents’ awareness regarding Ghana’s trade-related standards policy that restricts meat importation based on fat content remains fairly high and attitudes towards the restriction  is positive, over two decades after the ban was implemented. About half of those interviewed were aware of the restrictions, although more than half thought the public was not aware and majority felt enforcement done is ineffective. There does not seem to be a lot effort to raise public awareness about this policy. A search of newspaper coverage of the restrictions in the early 1990s, when the restrictions were passed, showed little coverage on the restrictions in general and the ban on turkey tail. The relatively high awareness, however, may also reflect regular enforcement efforts, which included destruction of 1452 cartons of turkey tail imports in Kumasi in 2010 [22]. The fact that majority (60%) thought the restriction in the long-term could improve health, prevent disease and reduce death was reassuring.

The findings also showed a good attitude of participants towards fatty meat and this may be indicative of a general good attitude towards healthy eating. It did seem that there has been some effort to sensitize the public on the effect of high fat intake and diet related chronic diseases by the level of knowledge/perception of these respondents. Also, close to 70% said they considered the fat content of the meat they bought and all seemed to know the negative effects of fat on health. This good attitude could also be due to the increasing trends of non communicable diseases in the country [4], which are associated with the diet and lifestyles of people [23] and which have gained some attention in the country. However, it was not clear whether awareness of these issues had increased over time because of lack of earlier studies to compare our findings.

Among the meat types, half of the population consumed local beef compared to less than 20% consuming the imported version, while half of the population consumed imported chicken regularly compared to less than a third consuming the local version. The disparity between the consumption of beef and chicken is mainly because imported chicken is commonly available and cheaper than its local counterpart, while local beef is more commonly available and cheaper than its imported counterpart. The very low consumption of turkey tails reported in this survey (less than 3%) indicates that the restrictions have been effective in significantly reducing availability and consumption of turkey tail, which was banned as a result of the restrictions. This is contrary to perception of respondents’ that the restriction is not well enforced. For all the meat types, with the exception of pork, participants predominantly preferred the local to the imported. The reasons given for the preference were better taste, access/cost, freshness, healthiness and religion. These factors, among others are known to influence people’s food habits and choice [24]. These factors seemed to be interwoven in their effect on preference and consumption. Irrespective of whether it is local or imported, some participants chose their meat based on health reasons and for this group, perception as to which version is more or less healthy informed their preference. But looking at the interplay of the factors, it seemed that availability and cheapness were the ultimate determinants and drivers of consumption of meat products. Majority of participants said imported meat products were more available and cheaper but our findings showed the vast majority of participants interviewed preferred the local to the imported meat types due to better taste and healthiness. Therefore, the fact that consumption of imported chicken, pork and processed meat were higher than the local types, although the local types were preferred suggests that availability/cheapness drove consumption, and confirms the worry about the food environment becoming more obesogenic [25]. This is why public health policies which restrict access to unhealthy diet, thereby making the food environment healthier as part of a package of interventions for prevention of non communicable diseases are recommended [14, 15].

This study could not evaluate the impact of the fatty meat restriction on consumption of meat products, since data on consumption before the restriction was not available. Little is known about the contribution of fatty meat to the Ghanaian diet. Moreover, although the participants of this study were all adults, majority were between 18 and 35 years old, and thus a large proportion were not old enough to share their experience before and after the implementation of the policy, nor was it possible to evaluate the impact of the policy on their consumption of meat types. However, the policy was passed in response to concerns that fatty meat, especially turkey tail and chicken feet were commonly imported and available on the market. The policy analysis paper earlier published explains the contexts and content of the policy [20]. The overall effect of the ban has been to reduce availability of specific ‘low quality’ meats in the Ghanaian food supply, namely turkey tails and chicken feet [20]. It was useful to understand the landscape 20 years after the policy implementation. Is fatty meat still a problem? What is the nutritional situation in Ghana now? Is it possible that this policy has helped to minimize this aspect of the nutrition transition? It was expected that the reduction in availability may have influenced consumption of fatty meat products. The very low consumption of turkey tail from the present study findings supports this.

## Conclusion

In conclusion this study showed appreciable awareness among Ghanaian adults regarding Ghana’s innovative trade policy, which restricts importation of meat and meat products based on fat content, two decades after implementation. There was a general good knowledge and positive attitude towards the restrictions and diet for health, reflected in preference for local meat types, which were considered to be healthier, tastier and fresher. However, consumption was driven more by availability and cheapness. Most respondents supported the restriction and were positive about the long-term positive effect of this policy on health and nutrition but thought the policy was not well enforced. There is therefore a call to improve enforcement and to implement other public health policies in order to make the food environment healthier and contribute towards preventing diet-related chronic diseases, which are on the rise in the country.

## Abbreviations

BMI: Body Mass Index
CEPS: Customs Excise and Preventive Services
CHD: Coronary Heart Disease
CHRPE: Committee on Human Research and Publications Ethics
CVD: Cardiovascular Disease
FFQ: Food Frequency Questionnaire
KATH: Komfo Anokye Teaching Hospital
KNUST: Kwame Nkrumah University of Science and Technology
SPSS: Statistical Package for Social Sciences
USA: United States of America
WHO: World Health Orgnisation
